# {Bis[4-(2-pyrid­yl)pyrimidin-2-yl] sulfide}dibromidocobalt(II)

**DOI:** 10.1107/S1600536808015821

**Published:** 2008-06-07

**Authors:** Cai-Yun Li, Xiao-Lan Tong, Tong-Liang Hu

**Affiliations:** a Department of Pharmaceutical Science, Tianjin Medical College, Tianjin 300222, People’s Republic of China; b Department of Chemistry, Nankai University, Tianjin 300071, People’s Republic of China

## Abstract

The title compound, [CoBr_2_(C_18_H_12_N_6_S)], is a mononuclear complex in which a twofold rotation axis passes through the Co and S atoms. The Co^II^ center is six-coordinated by four N atoms from one bis­[4-(2-pyrid­yl)pyrimidin-2-yl] sulfide (*L*) ligand and two bromide anions, forming an octa­hedral coordination geometry, where the four donor N atoms are located in the equatorial plane and the Br atoms occupy the axial positions. The sum of the bond angles around the Co atom in the equatorial plane is 360.5°, with the four N atoms and the central Co atom almost coplanar. In the crystal structure, the mononuclear units are linked by π–π stacking inter­actions (the inter­planar distances are 3.469 and 3.533 Å, and the corresponding centroid–centroid distances are 3.791 and 3.896 Å) into a three-dimensional supra­molecular network.

## Related literature

For related literature, see: de Faria *et al.* (2007 ▶); Teles *et al.* (2006 ▶); Li & Bu (2008 ▶); Bridson & Walker (1970 ▶).
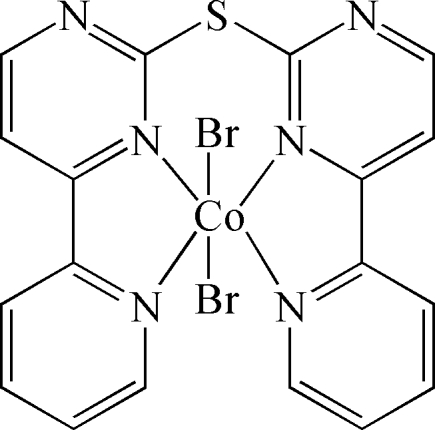


## Experimental

### 

#### Crystal data

[CoBr_2_(C_18_H_12_N_6_S)]
*M* *_r_* = 563.15
Monoclinic, 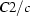

*a* = 15.191 (5) Å
*b* = 10.350 (4) Å
*c* = 13.338 (5) Å
β = 112.312 (5)°
*V* = 1940.0 (12) Å^3^
*Z* = 4
Mo *K*α radiation
μ = 5.13 mm^−1^
*T* = 294 (2) K
0.20 × 0.18 × 0.14 mm

#### Data collection

Bruker SMART 1000 CCD diffractometer
Absorption correction: multi-scan (*SADABS*; Bruker, 1998 ▶) *T*_min_ = 0.375, *T*_max_ = 0.489
5288 measured reflections
1970 independent reflections
1456 reflections with *I* > 2σ(*I*)
*R*_int_ = 0.036

#### Refinement

*R*[*F* ^2^ > 2σ(*F* ^2^)] = 0.028
*wR*(*F* ^2^) = 0.061
*S* = 1.04
1970 reflections
128 parameters
H-atom parameters constrained
Δρ_max_ = 0.39 e Å^−3^
Δρ_min_ = −0.36 e Å^−3^

### 

Data collection: *SMART* (Bruker, 1998 ▶); cell refinement: *SAINT* (Bruker, 1998 ▶); data reduction: *SAINT*; program(s) used to solve structure: *SHELXS97* (Sheldrick, 2008 ▶); program(s) used to refine structure: *SHELXL97* (Sheldrick, 2008 ▶); molecular graphics: *SHELXTL* (Sheldrick, 2008 ▶); software used to prepare material for publication: *SHELXTL*.

## Supplementary Material

Crystal structure: contains datablocks global, I. DOI: 10.1107/S1600536808015821/bq2083sup1.cif

Structure factors: contains datablocks I. DOI: 10.1107/S1600536808015821/bq2083Isup2.hkl

Additional supplementary materials:  crystallographic information; 3D view; checkCIF report

## Figures and Tables

**Table:** 

Br1—Co1	2.6178 (10)
Co1—N2	2.099 (2)
Co1—N1	2.125 (2)

**Table:** 

N2^i^—Co1—N2	96.18 (13)
N2—Co1—N1	78.04 (9)
N1—Co1—N1^i^	108.24 (13)
N2—Co1—Br1	86.87 (7)
N1—Co1—Br1	92.90 (7)

Symmetry code: (i) 
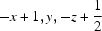
.

**Fig. 1.**
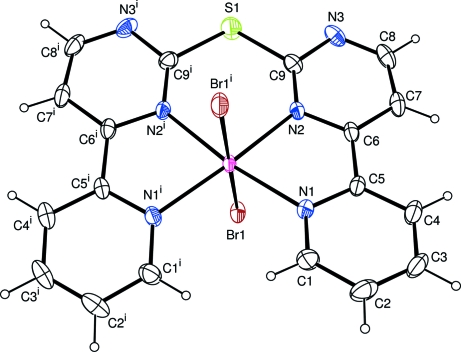
The molecular structure of (I). Displacement ellipsoids are drawn at the 30% probability level and H atoms are shown as small spheres of arbitrary radius [symmetry code: (i) -x + 1, y, -z + 1/2].

## supplementary crystallographic information

### Comment

Semirigid thioether ligands containing heterocycle with N-donors are gradually used in constructing coordination architectures with novel topologies and useful functions in recent years (de Faria *et al.*, 2007; Teles *et al.*, 2006). The pyridine and pyrimidine groups as normal heterocycle were used to incorporate with different mercapto units to form a series of bi- and multi-dentate ligands, which can adopt different conformations according to the different geometric requirements of metal centers when forming metal complexes (Li & Bu, 2008). Herein, we synthesized a semirigid thioether ligand bis[4-(pyridin-2-yl)pyrimidin-2-yl]sulfide (*L*) as well as its Co^II^ complex, [Co(C_18_H_12_N_6_S)Br_2_] (I), and report the crystal structure of this complex.

In the molecule of (I), (Fig. 1), the bond lengths and angles are generally within normal ranges (Bridson & Walker, 1970). The Co^II^ center is six-coordinated by four N atoms from one *L* ligand and two bromine ions, forming an octahedral coordination geometry. The bond angles N1—Co1—N2, N2—Co1—N2A, N2A—Co1—N1A and N1A—Co1—N1 are 78.04 (9), 96.18 (1), 78.04 (9) and 108.24 (1)°, respectively. The sum of these angles is 360.5 (2)°, suggesting that N1, N2, N1A, N2A and Co1 are almost in a plane. In the crystal structure of (I), the mononuclear units are interconnected by two π···π stacking interactions between the rings (N1/C1-C5) and (N2/N3/C6-C9) forming a three-dimensional supramolecular network.

### Experimental

The ligand bis[4-(pyridin-2-yl)pyrimidin-2-yl]sulfide (*L*) was synthesized according to the following method. Some amount of potassium 4-(pyridin-2-yl)pyrimine-2-thiolate was dissolved in the distilled water, and the dense hydrochloric acid was added drop by drop with slowly stirring. A lot of yellow precipitate appeared and then gradually disappear when the dense hydrochloric acid was continuously added. After complete disappearance of the yellow precipitate, the potassium hydroxide solution was added to the mixture until another kind of precipitate was obtained largely. The crude product was filtered and washed with the distilled water three times, and recrystallized with the mixture of chloroform and hexane (*v*/*v*=1:1), yield: 60%. The title coordination complex, (I), was synthesized according to the following method. A buffer layer of chloroform/methanol (*v*/*v*=2:1, 5 ml) was carefully layered over a chloroform solution (4 mL) of *L* (6.8 mg, 0.02 mmol). Then a solution of CoBr_2_ (4.4 mg, 0.02 mmol) in methanol (4 ml) was layered on the buffer layer. Red block crystals suitable for X-ray analysis were collected after five weeks, yield: 30%.

### Refinement

H atoms were included in calculated positions and treated in the subsequent refinement as riding atoms, with C—H = 0.93 and *U*_iso_(H) = 1.2*U*_eq_(C).

### Crystal data

**Table:**

[CoBr_2_(C_18_H_12_N_6_S)]	*F*_000_ = 1100
*M**_r_* = 563.15	*D*_x_ = 1.928 Mg m^−^^3^
Monoclinic, *C*2/*c*	Mo *K*α radiation λ = 0.71073 Å
Hall symbol: -c 2yc	Cell parameters from 2104 reflections
*a* = 15.191 (5) Å	θ = 2.6–26.1º
*b* = 10.350 (4) Å	µ = 5.13 mm^−^^1^
*c* = 13.338 (5) Å	*T* = 294 (2) K
β = 112.312 (5)º	Block, red
*V* = 1940.0 (12) Å^3^	0.20 × 0.18 × 0.14 mm
*Z* = 4	

### Data collection

**Table:**

Rigaku R-AXIS RAPID-S diffractometer	1970 independent reflections
Radiation source: fine-focus sealed tube	1456 reflections with *I* > 2σ(*I*)
Monochromator: graphite	*R*_int_ = 0.037
*T* = 294(2) K	θ_max_ = 26.3º
ω scans	θ_min_ = 2.4º
Absorption correction: multi-scan(SADABS; Bruker, 1998)	*h* = −18→16
*T*_min_ = 0.375, *T*_max_ = 0.489	*k* = −12→8
5288 measured reflections	*l* = −16→16

### Refinement

**Table:**

Refinement on *F*^2^	Secondary atom site location: difference Fourier map
Least-squares matrix: full	Hydrogen site location: inferred from neighbouring sites
*R*[*F*^2^ > 2σ(*F*^2^)] = 0.028	H-atom parameters constrained
*wR*(*F*^2^) = 0.061	*w* = 1/[σ^2^(*F*_o_^2^) + (0.0192*P*)^2^ + 2.0266*P*] where *P* = (*F*_o_^2^ + 2*F*_c_^2^)/3
*S* = 1.04	(Δ/σ)_max_ = 0.001
1970 reflections	Δρ_max_ = 0.39 e Å^−^^3^
128 parameters	Δρ_min_ = −0.36 e Å^−^^3^
Primary atom site location: structure-invariant direct methods	Extinction correction: none

### Special details

**Table:**

Geometry. All e.s.d.'s (except the e.s.d. in the dihedral angle between two l.s. planes) are estimated using the full covariance matrix. The cell e.s.d.'s are taken into account individually in the estimation of e.s.d.'s in distances, angles and torsion angles; correlations between e.s.d.'s in cell parameters are only used when they are defined by crystal symmetry. An approximate (isotropic) treatment of cell e.s.d.'s is used for estimating e.s.d.'s involving l.s. planes.
Refinement. Refinement of *F*^2^ against ALL reflections. The weighted *R*-factor *wR* and goodness of fit *S* are based on *F*^2^, conventional *R*-factors *R* are based on *F*, with *F* set to zero for negative *F*^2^. The threshold expression of *F*^2^ > σ(*F*^2^) is used only for calculating *R*-factors(gt) *etc*. and is not relevant to the choice of reflections for refinement. *R*-factors based on *F*^2^ are statistically about twice as large as those based on *F*, and *R*- factors based on ALL data will be even larger.

### Fractional atomic coordinates and isotropic or equivalent isotropic displacement parameters (Å^2^)

**Table:**

	*x*	*y*	*z*	*U*_iso_*/*U*_eq_	
Br1	0.47796 (2)	0.71842 (3)	0.43535 (3)	0.04031 (12)	
C1	0.3781 (2)	0.4680 (3)	0.1581 (3)	0.0462 (9)	
H1	0.4358	0.4248	0.1763	0.055*	
C2	0.2944 (3)	0.3973 (3)	0.1140 (3)	0.0540 (10)	
H2	0.2962	0.3085	0.1046	0.065*	
C3	0.2092 (3)	0.4609 (4)	0.0846 (3)	0.0515 (10)	
H3	0.1522	0.4153	0.0562	0.062*	
C4	0.2086 (2)	0.5924 (3)	0.0976 (3)	0.0425 (9)	
H4	0.1514	0.6372	0.0765	0.051*	
C5	0.29483 (19)	0.6578 (3)	0.1429 (2)	0.0297 (7)	
C6	0.30020 (19)	0.7994 (3)	0.1562 (2)	0.0302 (7)	
C7	0.2209 (2)	0.8785 (3)	0.1266 (3)	0.0415 (8)	
H7	0.1598	0.8441	0.1002	0.050*	
C8	0.2354 (2)	1.0094 (4)	0.1374 (3)	0.0457 (9)	
H8	0.1827	1.0636	0.1177	0.055*	
C9	0.3946 (2)	0.9792 (3)	0.2033 (2)	0.0353 (7)	
Co1	0.5000	0.71573 (5)	0.2500	0.02984 (15)	
N1	0.37930 (17)	0.5954 (2)	0.1755 (2)	0.0332 (6)	
N2	0.38904 (15)	0.8512 (2)	0.1956 (2)	0.0294 (6)	
N3	0.3215 (2)	1.0622 (3)	0.1749 (2)	0.0429 (7)	
S1	0.5000	1.07169 (11)	0.2500	0.0597 (4)	

### Atomic displacement parameters (Å^2^)

**Table:**

	*U*^11^	*U*^22^	*U*^33^	*U*^12^	*U*^13^	*U*^23^
Br1	0.02302 (16)	0.0472 (2)	0.0458 (2)	−0.00028 (14)	0.00748 (13)	0.00055 (16)
C1	0.044 (2)	0.0312 (19)	0.052 (2)	−0.0043 (15)	0.0054 (17)	−0.0006 (15)
C2	0.067 (3)	0.041 (2)	0.045 (2)	−0.0245 (19)	0.011 (2)	−0.0037 (17)
C3	0.042 (2)	0.060 (3)	0.045 (2)	−0.0292 (19)	0.0083 (18)	0.0007 (18)
C4	0.0269 (17)	0.060 (2)	0.038 (2)	−0.0124 (15)	0.0093 (15)	0.0018 (16)
C5	0.0214 (15)	0.0413 (18)	0.0251 (17)	−0.0055 (13)	0.0072 (13)	0.0007 (13)
C6	0.0209 (14)	0.043 (2)	0.0235 (16)	0.0028 (13)	0.0047 (12)	0.0025 (13)
C7	0.0206 (16)	0.061 (2)	0.040 (2)	0.0065 (15)	0.0089 (14)	0.0005 (17)
C8	0.0348 (19)	0.058 (2)	0.041 (2)	0.0238 (17)	0.0110 (16)	0.0041 (17)
C9	0.0355 (18)	0.0325 (17)	0.0349 (19)	0.0081 (14)	0.0100 (15)	0.0010 (14)
Co1	0.0159 (3)	0.0224 (3)	0.0438 (4)	0.000	0.0030 (2)	0.000
N1	0.0245 (13)	0.0318 (15)	0.0373 (16)	−0.0042 (10)	0.0051 (12)	0.0002 (11)
N2	0.0203 (12)	0.0295 (14)	0.0343 (15)	0.0032 (10)	0.0060 (11)	0.0024 (11)
N3	0.0433 (17)	0.0387 (16)	0.0437 (18)	0.0201 (13)	0.0130 (14)	0.0052 (13)
S1	0.0424 (7)	0.0221 (6)	0.0980 (12)	0.000	0.0079 (7)	0.000

### Geometric parameters (Å, °)

**Table:**

Br1—Co1	2.6178 (10)	C7—C8	1.371 (5)
C1—N1	1.338 (4)	C7—H7	0.9300
C1—C2	1.388 (5)	C8—N3	1.328 (4)
C1—H1	0.9300	C8—H8	0.9300
C2—C3	1.371 (5)	C9—N2	1.329 (4)
C2—H2	0.9300	C9—N3	1.339 (4)
C3—C4	1.373 (5)	C9—S1	1.764 (3)
C3—H3	0.9300	Co1—N2^i^	2.099 (2)
C4—C5	1.392 (4)	Co1—N2	2.099 (2)
C4—H4	0.9300	Co1—N1	2.125 (2)
C5—N1	1.353 (4)	Co1—N1^i^	2.125 (2)
C5—C6	1.475 (4)	Co1—Br1^i^	2.6178 (10)
C6—N2	1.359 (3)	S1—C9^i^	1.764 (3)
C6—C7	1.385 (4)		
			
N1—C1—C2	122.9 (3)	N2—C9—S1	126.2 (2)
N1—C1—H1	118.6	N3—C9—S1	107.2 (2)
C2—C1—H1	118.6	N2^i^—Co1—N2	96.18 (13)
C3—C2—C1	118.8 (3)	N2^i^—Co1—N1	171.98 (9)
C3—C2—H2	120.6	N2—Co1—N1	78.04 (9)
C1—C2—H2	120.6	N2^i^—Co1—N1^i^	78.04 (9)
C2—C3—C4	119.4 (3)	N2—Co1—N1^i^	171.98 (9)
C2—C3—H3	120.3	N1—Co1—N1^i^	108.24 (13)
C4—C3—H3	120.3	N2^i^—Co1—Br1	92.32 (7)
C3—C4—C5	119.1 (3)	N2—Co1—Br1	86.87 (7)
C3—C4—H4	120.4	N1—Co1—Br1	92.90 (7)
C5—C4—H4	120.4	N1^i^—Co1—Br1	87.82 (7)
N1—C5—C4	121.9 (3)	N2^i^—Co1—Br1^i^	86.87 (7)
N1—C5—C6	115.7 (2)	N2—Co1—Br1^i^	92.32 (7)
C4—C5—C6	122.4 (3)	N1—Co1—Br1^i^	87.82 (7)
N2—C6—C7	120.4 (3)	N1^i^—Co1—Br1^i^	92.90 (7)
N2—C6—C5	116.1 (2)	Br1—Co1—Br1^i^	178.78 (3)
C7—C6—C5	123.5 (3)	C1—N1—C5	117.8 (3)
C8—C7—C6	117.8 (3)	C1—N1—Co1	127.7 (2)
C8—C7—H7	121.1	C5—N1—Co1	114.53 (19)
C6—C7—H7	121.1	C9—N2—C6	116.6 (2)
N3—C8—C7	122.8 (3)	C9—N2—Co1	128.34 (19)
N3—C8—H8	118.6	C6—N2—Co1	114.81 (19)
C7—C8—H8	118.6	C8—N3—C9	115.7 (3)
N2—C9—N3	126.6 (3)	C9—S1—C9^i^	114.3 (2)
			
N1—C1—C2—C3	1.5 (6)	Br1—Co1—N1—C5	79.4 (2)
C1—C2—C3—C4	1.2 (6)	Br1^i^—Co1—N1—C5	−99.7 (2)
C2—C3—C4—C5	−1.6 (5)	N3—C9—N2—C6	−0.9 (4)
C3—C4—C5—N1	−0.7 (5)	S1—C9—N2—C6	−179.2 (2)
C3—C4—C5—C6	178.0 (3)	N3—C9—N2—Co1	−174.6 (2)
N1—C5—C6—N2	2.5 (4)	S1—C9—N2—Co1	7.1 (4)
C4—C5—C6—N2	−176.2 (3)	C7—C6—N2—C9	−0.2 (4)
N1—C5—C6—C7	179.6 (3)	C5—C6—N2—C9	177.1 (3)
C4—C5—C6—C7	0.9 (5)	C7—C6—N2—Co1	174.4 (2)
N2—C6—C7—C8	0.7 (5)	C5—C6—N2—Co1	−8.4 (3)
C5—C6—C7—C8	−176.4 (3)	N2^i^—Co1—N2—C9	−3.7 (2)
C6—C7—C8—N3	−0.1 (5)	N1—Co1—N2—C9	−178.0 (3)
C2—C1—N1—C5	−3.7 (5)	Br1—Co1—N2—C9	88.3 (2)
C2—C1—N1—Co1	176.2 (3)	Br1^i^—Co1—N2—C9	−90.8 (2)
C4—C5—N1—C1	3.3 (4)	N2^i^—Co1—N2—C6	−177.5 (2)
C6—C5—N1—C1	−175.5 (3)	N1—Co1—N2—C6	8.2 (2)
C4—C5—N1—Co1	−176.7 (2)	Br1—Co1—N2—C6	−85.46 (19)
C6—C5—N1—Co1	4.6 (3)	Br1^i^—Co1—N2—C6	95.45 (19)
N2—Co1—N1—C1	173.2 (3)	C7—C8—N3—C9	−0.9 (5)
N1^i^—Co1—N1—C1	−12.0 (2)	N2—C9—N3—C8	1.5 (5)
Br1—Co1—N1—C1	−100.6 (3)	S1—C9—N3—C8	−180.0 (2)
Br1^i^—Co1—N1—C1	80.4 (3)	N2—C9—S1—C9^i^	−3.8 (2)
N2—Co1—N1—C5	−6.8 (2)	N3—C9—S1—C9^i^	177.7 (3)
N1^i^—Co1—N1—C5	168.0 (2)		

Symmetry codes: (i) −*x*+1, *y*, −*z*+1/2.
